# Association Between Serum Creatinine Concentrations and Overall Survival in Patients With Colorectal Cancer: A Multi-Center Cohort Study

**DOI:** 10.3389/fonc.2021.710423

**Published:** 2021-10-07

**Authors:** Ming Yang, Qi Zhang, Guo-Tian Ruan, Meng Tang, Xi Zhang, Meng-Meng Song, Xiao-Wei Zhang, Kang-Ping Zhang, Yi-Zhong Ge, Han-Ping Shi

**Affiliations:** ^1^ Department of Gastrointestinal Surgery, Beijing Shijitan Hospital, Capital Medical University, Beijing, China; ^2^ Department of Clinical Nutrition, Beijing Shijitan Hospital, Capital Medical University, Beijing, China; ^3^ Department of Oncology, Capital Medical University, Beijing, China; ^4^ Laboratory of Beijing International Science and Technology Cooperation Base for Cancer Metabolism and Nutrition, Beijing, China; ^5^ Department of Cancer Radiotherapy and Chemotherapy, The Second Affiliated Hospital and Yuying Children’s Hospital of Wenzhou Medical University, Wenzhou, China

**Keywords:** colorectal cancer, serum creatinine, overall survival, renal function, muscle loss

## Abstract

**Background:**

Colorectal cancer (CRC) is one of the most common malignancies throughout the world, with high rates of morbidity and mortality. Previous studies reported that serum creatinine (Scr) concentrations were associated with overall survival (OS) in cancer patients, but little is known about the association between Scr and OS in patients with CRC. This study investigated the relationship between Scr concentrations and OS in patients with CRC and examined possible effect modifiers.

**Methods:**

A retrospective cohort, including 1,733 patients with CRC, was established from a multi-center clinical study. Patients were divided into low (<71 μmol/L in men or <59 μmol/L in women), normal (71-104 μmol/L in men or 59-85 μmol/L in women) and high (>104 μmol/L in men or >85 μmol/L in women) Scr groups. Cox regression analysis was used to examine association between Scr concentrations and OS. Stratified (subgroup) analyses were used to examine men and women separately. Interaction tests were used to evaluate associations between each variable and OS, as well as possible interactions of these variables with Scr levels. Cross-classified analyses were used only in men.

**Results:**

Patients with low [hazard ratio (HR) = 1.43, 95% confidence interval (CI) = 1.19-1.72; *P* < 0.001] or high (HR = 1.89, 95% CI = 1.36-2.63; *P* < 0.001) Scr level had a significantly lower OS than patients with normal Scr levels. Significant interactions with Scr concentrations were observed for body mass index (*P* for interaction = 0.019) in men.

**Conclusion:**

Low or high Scr concentration is associated with significantly lower OS in patients with CRC. Future study is warranted to investigate the underlying mechanism.

## Introduction

Colorectal cancer (CRC) is one of the most common malignancies throughout the world. Patients with CRC have the fourth highest morbidity rate and the second highest mortality rate (1). Although many treatment strategies have been developed for CRC, such as chemotherapy, surgery, radiation, targeted therapy, and immunotherapy, nearly 900,000 people worldwide died from CRC in 2018. Negative impacts on society from CRC include decreased mental health and quality of life, as well as an increased economic and medical burden (1). Numerous methods have been developed to accurately evaluate prognosis and improve survival of cancer patients. Although several studies, most of which included less than 400 patients, stressed the importance of clinical parameters such as performance status, elevated lactate dehydrogenase levels, white blood cell count, serum albumin, liver trans-aminases, hemoglobin, platelets, carcinoembryonic antigen, carbohydrate antigen19-9, pathological grading, and location of the primary tumor, there is no general consensus for considering each of these parameters as a valid and reliable prognostic factor (2–7). Therefore, there is an urgent need to develop new methods to identify important and modifiable prognostic factors to improve survival in patients with CRC.

Serum creatinine (Scr) is a chemical waste product produced by muscle metabolism that is normally excreted in the urine. Over the past few years, Scr concentrations have been investigated as a prognostic marker for various cancers. Willegger et al. studied 132 patients undergoing sarcoma resection and found that Scr could be used as a predictive biomarker for disease-specific outcomes in myofibroblastic and fibroblastic sarcomas (8). In a retrospective cohort study, Scr was an independent predictor of overall survival (OS) in 498 patients with primary epithelial ovarian cancer (9). Życzkowski et al. found that Scr may be useful for estimating the 5-year survival of patients with renal cell carcinoma (10). Similarly, Panotopoulos et al. found that Scr was highly associated with disease-specific survival in 84 patients with liposarcoma (11).

Based on the studies outlined above, Scr concentrations may be useful for predicting survival outcomes in various cancers. In this study, we evaluated the association between Scr levels and OS and investigated possible effect modifiers in patients with CRC.

## Materials and Methods

### Study Population

**Figure 1:**
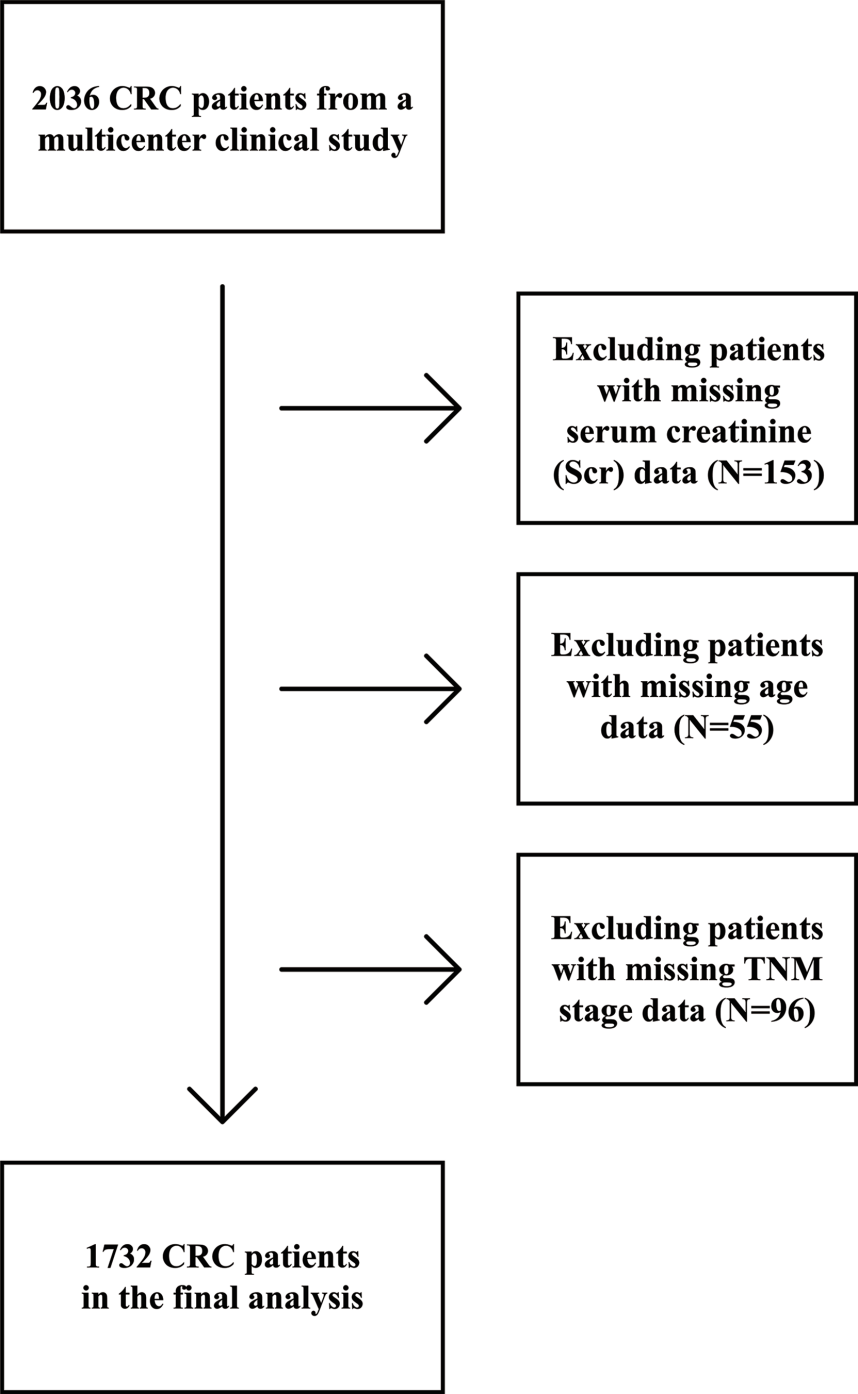
Flow chart of study participants with colorectal cancer (CRC) from a multi-center clinical trial.

A total of 2036 patients with CRC in China were enrolled from a multicenter retrospective cohort at 14 hospitals from January 2012 to December 2019. All patients were selected based on strict inclusion and exclusion criteria: (1) age ≥ 18 years; (2) a pathological diagnosis of CRC; (3) able and willing to provide written informed consent; and (4) conscious with no communication disorders. Some patients were considered to be a single case even though they may have been hospitalized more than two times during the study. Ninety-three patients who had missing data for one or more critical variables [153 patients missing Scr concentrations, 54 patients with no age data, and 96 patients missing TNM stage (T for tumor; N for node; M for metastasis)] were excluded from this study (**Figure 1**). The trial was approved by the Medical Ethical Review Committees/Institutional Review Boards of the registration hospitals mentioned above and was conducted in accordance with the Declaration of Helsinki. The trial was registered with the Chinese Clinical Trial Registry (http://www.chictr.org.cn) with registration number ChiCTR1800020329.

### Patient Characteristics and Outcomes

The following demographic and clinicopathological data were collected: Scr levels, age, sex, alcohol consumption, smoking status, body mass index (BMI), TNM stage, and chemotherapy (12). Based on a previous study about the reference interval of Scr levels in Chinese common population, patients were divided into low (<71 μmol/L in men or <59 μmol/L in women), normal (71-104 μmol/L in men or 59-85 μmol/L in women) and high (>104 μmol/L in men or >85 μmol/L in women) Scr groups (13). Pathological staging followed the American Joint Committee on Cancer (AJCC) TNM staging system, 8th edition (14). The Scr test was standardized in China with the same protocol and reference range nationwide. Hence, there was no difference in the normal range of Scr across provinces. Patient deaths due to the progression of CRC were defined as the primary end point.

### Statistical Analysis

All data are expressed as the mean ± standard deviation or simple percentages as appropriate. Baseline characteristics were analyzed using Chi-square tests or Fisher’s exact tests, as appropriate for categorical variables. Student’s t-tests were used for continuous variables with normal distributions and Mann‐Whitney tests were used for continuous variables with non-normal distributions. Hazard ratios (HR) were calculated using Cox regression analysis.

Association between Scr concentrations and OS in patients with CRC was estimated by multivariable Cox regression analysis with adjustment for sex (for all patients), age, alcohol consumption, smoking status, BMI, TNM stage and chemotherapy. Stratified (subgroup) analyses were performed to examine male and female patients separately. Interaction tests were performed to evaluate associations between each variable and OS, as well as possible interactions among these variables and Scr levels. Cross-classified analyses were performed only for men. A *P*-value <0.05 was considered statistically significant. All analyses were performed using R (version 4.0.1) and SPSS (version 26.0) software.

## Results

### Study Population and Baseline Characteristics

This study included 1,733 patients with CRC from the multi-center clinical study cohort with complete data (**Figure 1**). Sex-specific Scr concentrations are presented in **Table 1**. Compared with patients with normal Scr levels, more smokers, less men and drinkers, older age and lower chemotherapy proportion was observed in patients with high Scr levels, less drinkers and higher TNM stage in patients with low Scr levels.

**Table 1:** Characteristics of all patients with different Scr levels.

Characteristics	Patients with low Scr levels (n = 701)*^a^*	Patients with normal Scr levels (n = 922)*^b^*	Patients with high Scr levels (n = 109)*^c^*	*P* value
**Sex*^d^* (man)**	350 (49.9)	617 (66.9)	67 (61.5)	<0.001
**Age in years*^e^***	58.91 (11.46)	59.22 (12.32)	64.62 (12.58)	<0.001
**Smoking status*^d,f^* (Yes)**	239 (34.1)	385 (41.8)	47 (43.1)	0.005
**Alcohol consumption*^d,g^* (Yes)**	140 (20.0)	187 (20.3)	17 (15.6)	0.508
**BMI*^e^* (kg/m^2^)**	22.17 (3.37)	22.79 (3.30)	23.05 (3.03)	<0.001
**TNM stage*^d^***				0.255
**I or II**	222 (31.7)	327 (35.5)	39 (35.8)	
**III or IV**	479 (68.3)	595 (64.5)	70 (64.2)	
**Chemotherapy*^d^* (Yes)**	382 (54.5)	511 (55.4)	56 (51.4)	0.709

Scr, serum creatinine; BMI, body mass index.

**^a^** Low: Scr levels <71 μmol/L in men and <59 μmol/L in women.

**^b^** Normal: Scr levels ≥71 and ≤104 μmol/L in men and ≥59 and ≤85 μmol/L in women.

**^c^** High: Scr levels >104 μmol/L in men and >85 μmol/L in women.

**^d^** Categorical variables are presented as number (percentage).

**^e^** Continuous variables are presented as mean (standard deviation).

**^f^** The standard is to smoke more than 20 cigarettes in a lifetime.

**^g^** The standard is regular drinking in the past year.

### Association Between Scr Concentrations and OS in Patients With CRC

**Figure 2:**
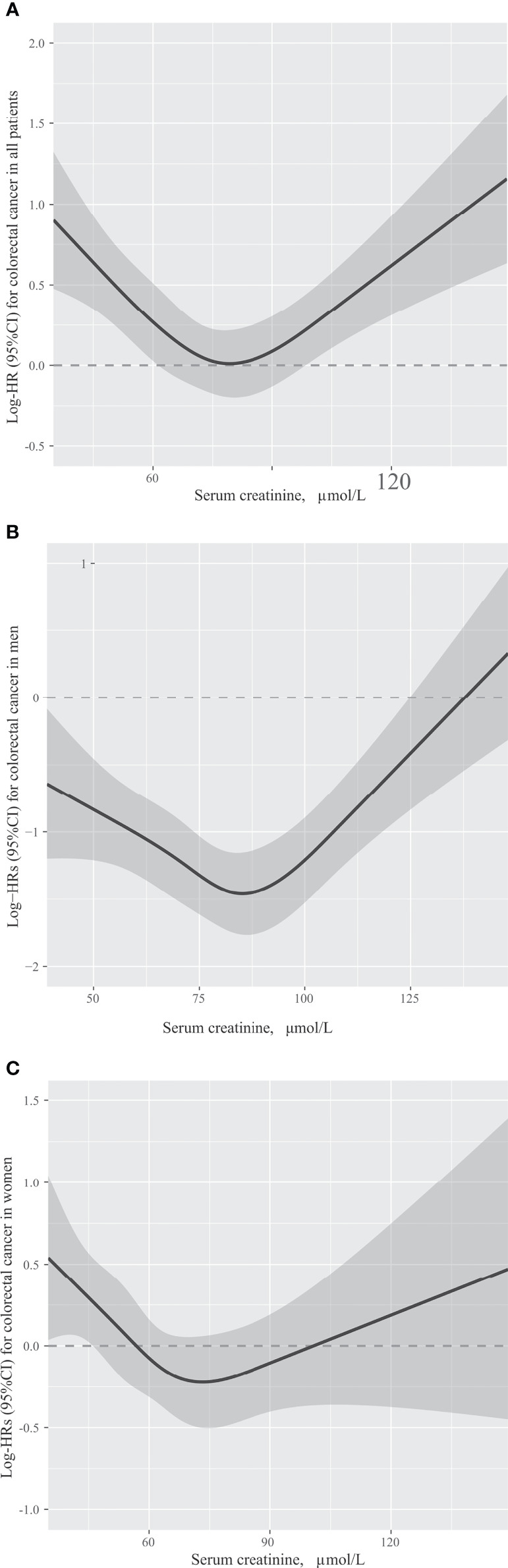
The association between serum creatinine concentrations and overall survival in patients with colorectal cancer in all patients **(A)**, men **(B)** and women **(C)**. Models were adjusted by sex (only in all patients), age, TNM stage, smoking status, alcohol consumption, body mass index and chemotherapy.

The association between Scr concentrations and OS in patients with CRC is presented in **Figure 2**. Patients with low Scr levels (HR = 1.37, 95% CI = 1.14-1.64; *P* = 0.001) or high Scr levels (HR = 1.78, 95% CI = 1.28-2.46; *P* = 0.001) had a significantly lower OS than patients with normal Scr levels (**Table 2**). An association between low (HR = 1.39, 95% CI = 1.16-1.67; *P <*0.001) or high (HR = 1.81, 95% CI = 1.30-2.51; *P <*0.001) Scr concentrations and OS could be observed after adjusting for sex, age and TNM stage. A significant association between low (HR = 1.43, 95% CI = 1.19-1.72; *P <*0.001) or high (HR = 1.89, 95% CI = 1.36-2.63; *P <*0.001) Scr concentration and OS in patients with CRC was detected after adjusting for potential covariates: sex, age, alcohol consumption, smoking status, BMI, TNM stage and chemotherapy.

**Table 2:** Association between Scr concentrations and OS patients with CRC.

Characteristics	Patients (n)	Crude HR (95% CI)	*P* value	Adjusted HR (95% CI)*^a^*	*P* value	Adjusted HR (95% CI)*^b^*	*P* value
**Scr of all patients**							
Low*^c^*	701	1.37 (1.14, 1.64)	0.001	1.39 (1.16, 1.67)	<0.001	1.43 (1.19, 1.72)	<0.001
Normal*^d^*	922	Reference		Reference		Reference	
High*^e^*	109	1.78 (1.28, 2.46)	0.001	1.81 (1.30, 2.51)	<0.001	1.89 (1.36, 2.63)	<0.001
**Scr of men**							
Low*^c^*	350	1.35 (1.07, 1.70)	0.011	1.26 (1.00, 1.59)	0.050	1.32 (1.05, 1.66)	0.019
Normal*^d^*	617	Reference		Reference		Reference	
High*^e^*	67	1.93 (1.29, 2.87)	0.001	1.96 (1.31, 2.93)	0.001	2.06 (1.37, 3.09)	<0.001
**Scr of women**							
Low*^c^*	351	1.50 (1.11, 2.03)	0.009	1.62 (1.19, 2.20)	0.002	1.62 (1.19, 2.21)	0.002
Normal*^d^*	305	Reference		Reference		Reference	
High*^e^*	42	1.64 (0.93, 2.87)	0.085	1.64 (0.93, 2.89)	0.086	1.71 (0.97, 3.01)	0.063

Scr, serum creatinine; OS, overall survival; CRC, colorectal cancer; HR, hazard ratio; CI, confidence interval.

^a^Models were adjusted by sex (only in all patients), age, TNM stage.

^b^Models were adjusted by sex (only in all patients), age, TNM stage, smoking status, alcohol consumption, body mass index and chemotherapy.

^c^Low: Scr levels <71 μmol/L in men and <59 μmol/L in women.

^d^Normal: Scr levels ≥71 and ≤104 μmol/L in men and ≥59 and ≤85 μmol/L in women.

^e^High: Scr levels >104 μmol/L in men and >85 μmol/L in women.

### Sex-Specific Associations Between Scr Concentrations and OS in Patients With CRC

Results of the adjusted sex-specific subgroup analysis of OS in patients with CRC were similar to results of the analysis that included all patients. Men with low Scr levels (HR = 1.35, 95% CI = 1.07-1.70; *P* = 0.011) or high Scr levels (HR = 1.93, 95% CI = 1.29-2.87; *P* = 0.001) had a significantly lower OS than men with normal Scr levels (**Table 2**). Compared with women with normal Scr concentrations, lower OS (HR = 1.50, 95% CI = 1.11-2.03; *P* = 0.009) was observed in women with low Scr concentrations (**Table 2**). After adjusting for age and TNM stage, the association between low Scr concentrations and OS was still stable either in men or women (men: HR = 1.26, 95% CI = 1.00-1.59; *P* = 0.050; women: HR = 1.62, 95% CI = 1.19-2.20; *P* = 0.002). Although the results were not statistically significant after adjusting for age and TNM stage in women with high Scr levels, men with high Scr levels still had poor OS (HR = 1.96, 95% CI = 1.31-2.93; *P* = 0.001). In men and women with low Scr levels, adjusting for age, alcohol consumption, smoking status, BMI, TNM stage and chemotherapy did not significantly alter the results (men: HR = 1.32, 95% CI = 1.05-1.66; *P* = 0.019; women: HR = 1.62, 95% CI = 1.19-2.21; *P* = 0.002). Although the results were not statistically significant after adjusting for potential covariates in women with high Scr levels (HR = 1.71, 95% CI = 0.97-3.01; *P* = 0.063), men with high Scr levels still had poor OS (HR = 2.06, 95% CI = 1.37-3.09; *P <*0.001).

### Sensitivity Analyses

The first sensitivity analysis examined association between normal Scr concentrations and CRC mortality in the overall study population, and separately in men and women. Whether Scr concentration was used as a continuous variable or divided into four quartile groups, no significant correlation between Scr concentrations and OS in patients with CRC was observed (**Supplemental Table 1**).

In the second sensitivity analysis, association between Scr concentrations and OS was analyzed in patients that were divided into two groups, those who died within one year from baseline and those who died after more than one year from baseline (**Supplemental Tables 2, 3**). The trend between low Scr concentrations and lower OS was observed in all patients (HR = 1.44 95% CI = 1.10-1.90; *P* = 0.009) and men (HR = 1.42, 95% CI = 1.01-2.01; *P* = 0.049) who died within one year after adjusting for age and TNM stage. A significant association between low Scr concentration and OS in all patients who died within one year (HR = 1.42, 95% CI = 1.08-1.88; *P* = 0.013) was detected after adjusting for potential covariates. In patients who died after more than one year from baseline, no significant correlation between Scr concentrations and OS in patients with CRC was observed.

### Analyses Stratified by Potential Effect Modifiers

**Figure 3:**
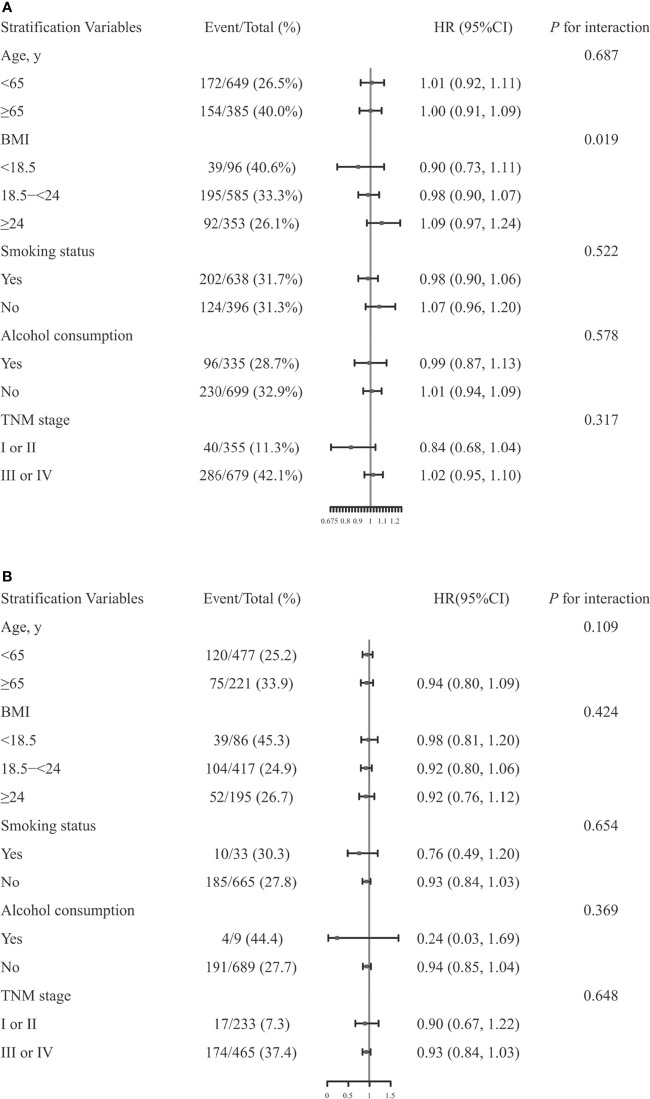
The association between serum creatinine (Scr) concentrations and overall survival (OS) in men **(A)** or women **(B)** with colorectal cancer in various subgroups. Models were adjusted by age, TNM stage, smoking status, alcohol consumption, body mass index (BMI) and chemotherapy, but were not adjusted for the stratifying variable.

Stratified analyses were performed to assess association between Scr concentrations and OS in patients with CRC in various subgroups (**Figure 3**). Due to sex differences, men and women were analyzed separately.

Significant differences were observed among men based on BMI (<18.5 *vs.* 18.5-<24 *vs.* ≥24, *P* for interaction = 0.019) (15). However, none of the other variables, including age (<65 *vs.* ≥65, *P* for interaction = 0.687), smoking status (Yes *vs.* No, *P* for interaction = 0.522), alcohol consumption (Yes *vs.* No, *P* for interaction = 0.578), TNM stage (I or II *vs.* III or IV, *P* for interaction = 0.317), modified association between Scr concentrations and OS in men with CRC. In women, there were no significant interactions for any of the subgroups (all *P* for interactions >0.05).

### Survival and Cross-Classified Analysis

**Figure 4:**
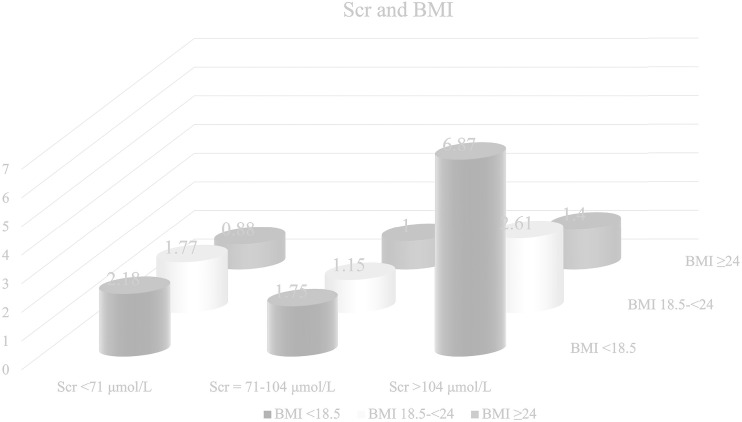
Hazard ratio for the interaction between body mass index (BMI) and serum creatinine (Scr) levels, respectively. Model was adjusted by age, TNM stage, smoking status, alcohol consumption and chemotherapy.

Kaplan-Meier curves showed that patients with high or low Scr levels and BMI <18.5 had significantly lower OS than those with normal Scr levels (*P* = 0.001) and BMI ≥24 (*P <*0.001) (**Supplemental Figure 1**). We then cross-classified BMI with Scr to understand the differential effects of these two variables (**Figure 4**). Patients with high Scr levels and BMI <18.5 had a nearly 7-fold increased risk of death (HR = 6.87, 95% CI = 1.64-28.86) compared with those with normal Scr levels and BMI ≥24.

## Discussion

CRC is a common malignant tumor in the gastrointestinal tract, and the prognosis of patients with CRC is poor (16). The incidence and mortality attributable to CRC has increased worldwide over the last several decades (17). Although biomarkers associated with OS in patients with CRC may be moderately predictive of survival, additional research and evaluation are needed to improve their predictive utility (18–21). In this study, we evaluated association between Scr levels and OS in patients with CRC because Scr has been reported to be predictive of prognosis in many cancers (8–11). Our study revealed a correlation between Scr concentrations and OS in patients with CRC, which was consistent with previous studies in other cancers.

In this retrospective multi-center clinical study with relatively large variability across patients with CRC, we found that a high or low Scr concentration was associated with significantly lower OS in comparison to patients with normal concentrations. Furthermore, our study suggests that association between Scr and OS in patients with CRC may be modified by BMI in men.

It is usually considered that Scr levels are dependent on the balance between its production and excretion (22). Creatinine is formed as a result of the nonenzymatic dehydration of muscle creatine (23). Creatinine production relies on the size of the creatine pool, which is determined by total muscle mass and dietary intake of meat (24). Creatinine is a small molecule that is normally filtered through the glomerulus and secreted by renal tubular cells, while it is rarely absorbed in the renal tubules (23).

In our study, high or low Scr level beyond the normal range was associated with significantly lower OS, which may imply a disruption of the balance mentioned above. Therefore, we discuss these observations from two aspects: (1) the relationship between low Scr concentrations and lower OS of patients with CRC, and (2) the relationship between high Scr concentrations and lower OS of patients with CRC.

The association between low Scr concentrations and lower OS in patients with CRC may be attributed to a high filtration rate and/or a low production volume of creatinine (23, 24). A previous study reported that augmented renal clearance occurred in critically ill patients characterized by increased creatinine clearance, and such a high creatinine clearance rate may be responsible for lower OS (24). Furthermore, low muscle mass and reduced food intake may be responsible for the low production volume of creatinine in patients with CRC (24). Previous studies have also shown that patients with CRC are often accompanied by loss of appetite and reduced food intake (25). At the same time, due to the systemic inflammation, loss of appetite and serious energy cost of the patients with malignant tumor, their muscle mass would decrease in varying degrees at the early stage of cancer (26). Low muscle mass and reduction in food intake were suggested to closely associate with lower OS in patients with CRC (27–30). Based on these study and our results, the association between low Scr levels and low OS may reflect critical condition, low food intake and low muscle mass. Therefore, the level of serum creatinine may predict prognosis and guide clinical treatment of CRC. However, further studies are warranted to prove our theory and explore possible mechanisms.

A decrease in excretion may explain the relationship between high Scr concentrations and low OS in patients with CRC. Previous studies have shown that high Scr concentrations may be an indicator of severe renal damage (24). Studies have shown that the systemic inflammatory state of patients with CRC, the decrease of drinking water caused by loss of appetite, and the disrupted electrolyte balance caused by diarrhea induced by intestinal environmental changes can lead to the damage of renal function (31–35). Several studies have reported that patients with CRC with abnormal renal function have a significantly lower OS than those with normal renal function, which was consistent with our study (36, 37). Moreover, patients with CRC were at risk for damage to renal function and complications from some anticancer treatments (38). Conversely, a decline in renal function may jeopardize a patient’s eligibility for further cancer treatment, increase toxicity, prevent the delivery of chemotherapy, and exclude patients from clinical trials. These conditions may further aggravate complications and the deterioration of a patient’s physical condition, resulting in a further decline of renal function (38). This vicious cycle has the potential to accelerate disease progression and decrease OS in patients with CRC. In addition to renal excretion, intestinal microbial metabolism is an important Scr excretion pathway (39–41). Previous studies have shown that the intestinal microbiota of patients with CRC was significantly different from that in healthy people, which might affect creatinine metabolism and lead to an increase in Scr levels (34, 41). Previous research has reported that the intestinal microbiota has a profound impact on the prognosis of patients with CRC (33). Specifically, changes in the abundance and/or composition of some intestinal microbiota may decrease OS in patients with CRC (33). Other potential routes of extrarenal creatinine excretion, such as perspiration and fecal loss, were insignificant (24). Therefore, serum creatinine can be used as a prognostic factor for patients with CRC and an indicator of potential clinical strategies.

In this study, we performed two sensitivity analyses. In the first analysis, we found no significant correlation between normal Scr concentrations and OS in patients with CRC overall, in men only, or in women only, consistent with our clinical experience. In the second analysis, low OS in patients who died within one year was significantly associated with low Scr concentrations but was not associated with high Scr concentrations in the overall patient population. In previous studies, researchers found that patients with less muscle had a shorter survival time (31). This may prompt us that we should start nutritional support treatment at the early stage of CRC, rather than when the patient is complicated with cachexia or sarcopenia.

Finally, we examined possible effect modifiers and found that BMI had significant interaction with Scr. We observed that BMI <18.5 was associated with lower OS in patients with CRC, and that this variable had significant interactive effects with high or low Scr concentrations. Patients with BMI <18.5 and high Scr levels had a nearly 7-fold increased risk of death compared with those with BMI ≥24 and normal Scr concentrations. Previous research suggests that low BMI may be due to severe malnutrition, inadequate intake and systemic inflammatory response, and these factors can also lead to the damage of renal function (35, 42). Meanwhile, high Scr levels also suggest renal insufficiency (24). Therefore, elevated serum creatinine levels in patients with low BMI could usually indicate more severe renal impairment and poorer OS than the patients in single condition. In conclusion, enough enteral and parenteral nutrition support and anti-inflammatory treatment to improve the factors leading to BMI reduction would benefit OS of patients with CRC.

There were several limitations of our study. Our laboratory data were obtained from common laboratory tests and were limited in scope. In the future, we plan to recruit more patients to increase our statistical power and to increase the number of variables in our dataset by examining additional less-common factors to explore their relationship with the occurrence and survival for various cancers.

## Conclusions

In conclusion, in this multi-center clinical study, we found that high or low Scr concentrations were associated with significantly lower OS in patients with CRC. Future study is warranted to explore the potential mechanism behind the association.

## Data Availability Statement

The raw data supporting the conclusions of this article will be made available by the authors, without undue reservation.

## Ethics Statement

The studies involving human participants were reviewed and approved by Medical Ethics Committee of First Affiliated Hospital of Sun yat-sen University. The patients/participants provided their written informed consent to participate in this study.

## Author Contributions

HPS: Conceptualization and methodology. MY: Data curation and writing-original draft preparation. QZ: Visualization, investigation, and data curation. GTR: Software. MT: Validation and visualization. XZ: Writing-reviewing and editing. MMS: Writing-reviewing and editing. XWZ: Supervision and investigation. KPZ: Software. YZG: Supervision and investigation.

## Funding

This work was supported by the National Key Research and Development Program (2017YFC1309200).

## Conflict of Interest

The authors declare that the research was conducted in the absence of any commercial or financial relationships that could be construed as a potential conflict of interest.

## Publisher’s Note

All claims expressed in this article are solely those of the authors and do not necessarily represent those of their affiliated organizations, or those of the publisher, the editors and the reviewers. Any product that may be evaluated in this article, or claim that may be made by its manufacturer, is not guaranteed or endorsed by the publisher.

## Supplementary Material

Supplementary Figure 1 Kaplan-Meier curves of serum creatinine (Scr) **(A)** and body mass index (BMI) **(B)**.
