# Supplementary figures and images for: Association Between Serum Creatinine Concentrations and Overall Survival in Patients With Colorectal Cancer: A Multi-Center Cohort Study
Source: Front Oncol. 2021 Oct 7;11:710423. doi: 10.3389/fonc.2021.710423 (PMC8529284; doi:10.3389/fonc.2021.710423)

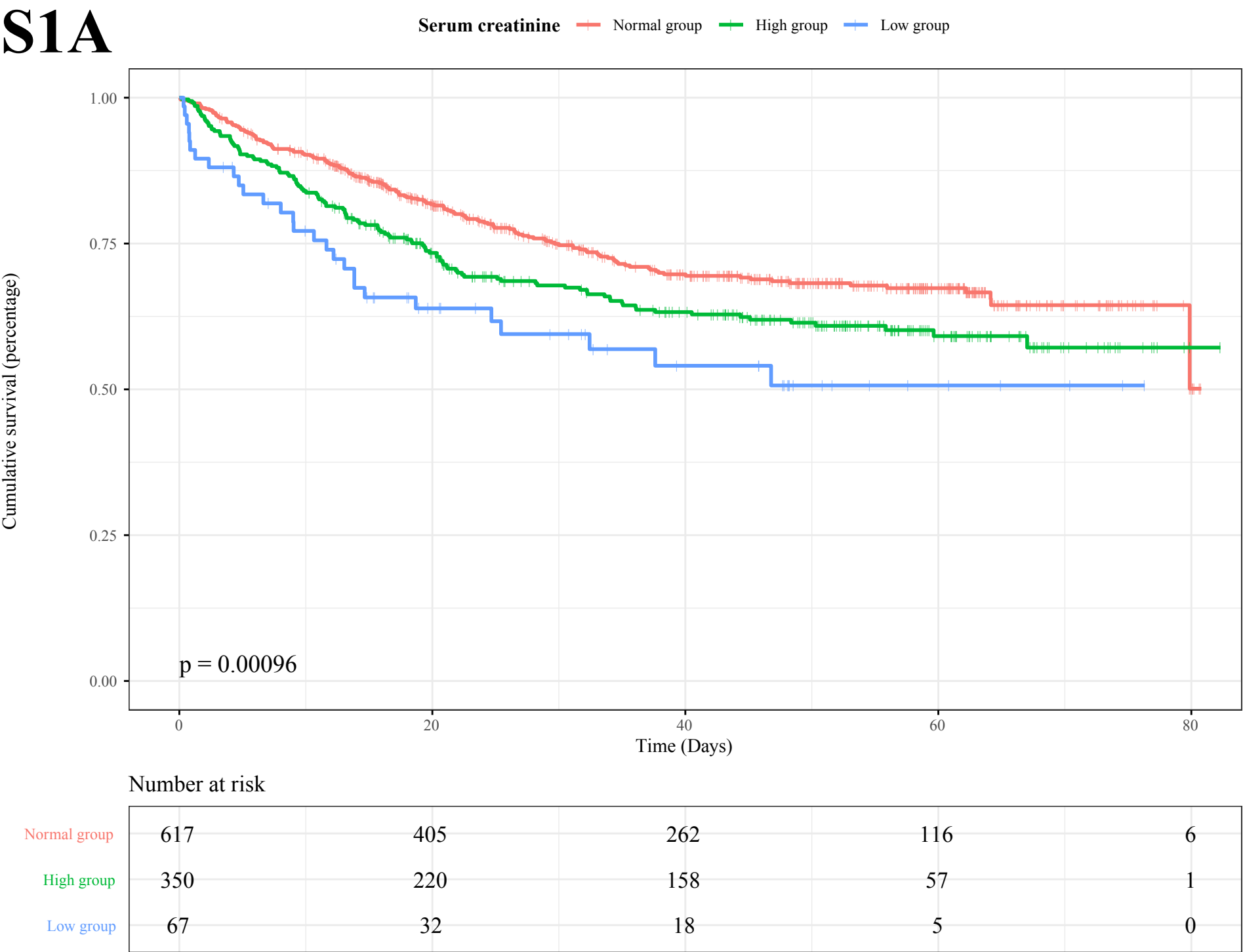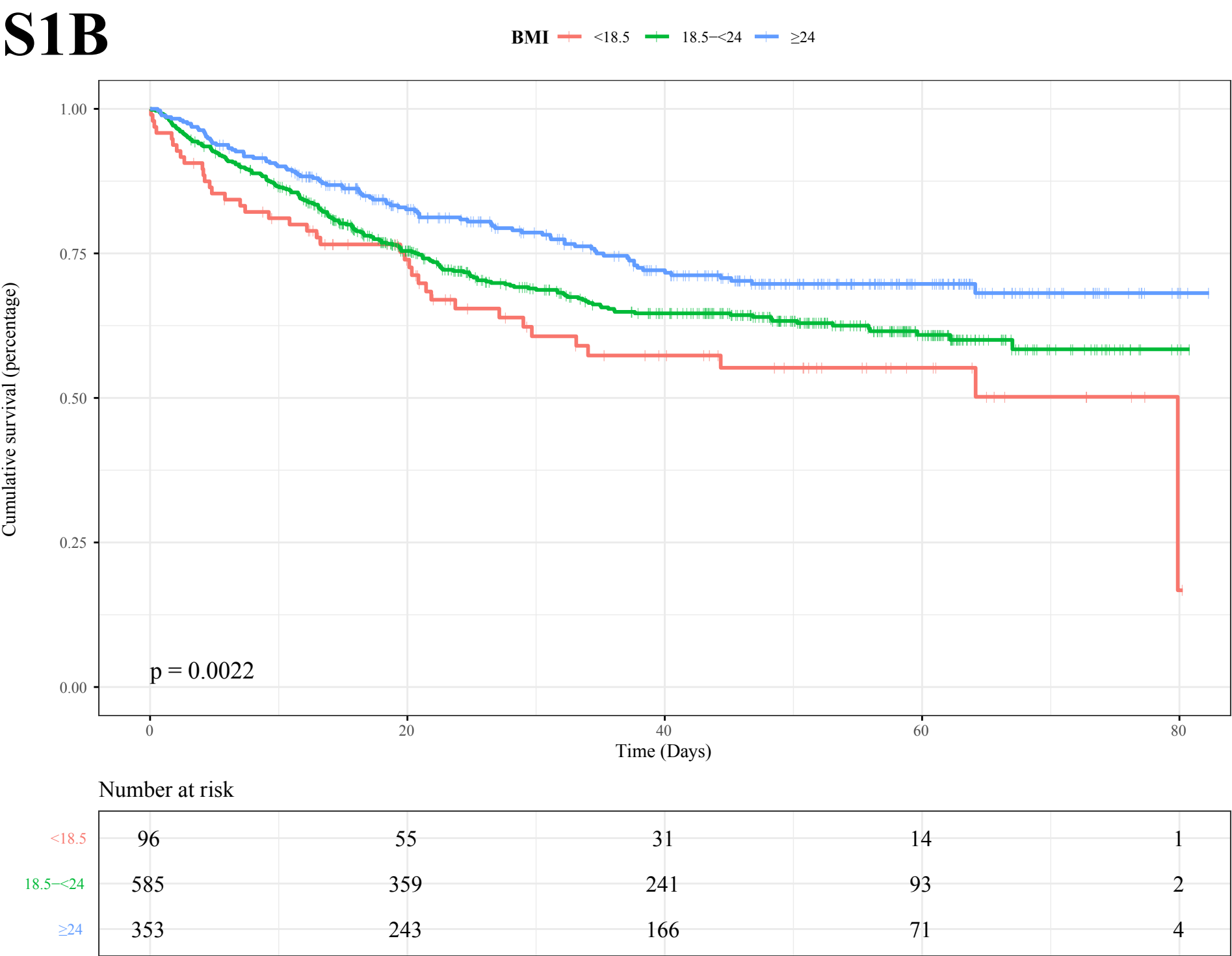

Supplement: Supplementary Figure 1 — Kaplan-Meier curves of serum creatinine (Scr) (A) and body mass index (BMI) (B). [file DataSheet_1.pdf]
