# Supplementary material for: Association Between Serum Creatinine Concentrations and Overall Survival in Patients With Colorectal Cancer: A Multi-Center Cohort Study
Source: Front Oncol. 2021 Oct 7;11:710423. doi: 10.3389/fonc.2021.710423 (PMC8529284; doi:10.3389/fonc.2021.710423)
Supplement: Supplementary file 2 [file Table_1.docx]

**Supplemental table 1 Association between normal Scr concentrations and OS in patients with CRC.**

| Characteristics (Continuous/Quartiles) | Patients (n) | Adjusted HR (95% CI) *^a^* | *P* value | Adjusted HR (95% CI) *^b^* | *P* value |
| --- | --- | --- | --- | --- | --- |
| Normal Scr of all patients |  |  |  |  |  |
| Continuous | 922 | 0.93 (0.80, 1.09) | 0.388 | 0.95 (0.81, 1.12) | 0.557 |
| Quartiles 1 | 230 | Reference |  | Reference |  |
| Quartiles 2 | 242 | 1.15 (0.73, 1.81) | 0.558 | 1.22 (0.78, 1.92) | 0.386 |
| Quartiles 3 | 220 | 1.07 (0.66, 1.75) | 0.784 | 1.16 (0.71, 1.89) | 0.550 |
| Quartiles 4 | 230 | 0.87 (0.52, 1.48) | 0.616 | 0.95 (0.56, 1.61) | 0.853 |
| Normal Scr of men |  |  |  |  |  |
| Continuous | 617 | 0.89 (0.74, 1.06) | 0.188 | 0.91 (0.76, 1.09) | 0.318 |
| Quartiles 1 | 168 | Reference |  | Reference |  |
| Quartiles 2 | 150 | 1.43 (0.96, 2.14) | 0.079 | 1.47 (0.98, 2.19) | 0.061 |
| Quartiles 3 | 148 | 0.72 (0.46, 1.13) | 0.150 | 0.72 (0.46, 1.13) | 0.155 |
| Quartiles 4 | 151 | 0.90 (0.59, 1.36) | 0.617 | 0.96 (0.63, 1.47) | 0.854 |
| Normal Scr of women |  |  |  |  |  |
| Continuous | 305 | 1.11 (0.81, 1.52) | 0.520 | 1.11 (0.80, 1.54) | 0.515 |
| Quartiles 1 | 77 | Reference |  | Reference |  |
| Quartiles 2 | 83 | 0.85 (0.42, 1.72) | 0.653 | 0.83 (0.41, 1.68) | 0.607 |
| Quartiles 3 | 79 | 0.94 (0.49, 1.83) | 0.865 | 0.92 (0.47, 1.79) | 0.798 |
| Quartiles 4 | 66 | 1.01 (0.52, 1.95) | 0.986 | 1.03 (0.53, 2.02) | 0.924 |

Notes:

Abbreviations: Scr, serum creatinine; OS, overall survival; CRC, colorectal cancer; HR, hazard ratio; CI, confidence interval.

***^a^*** Models were adjusted by sex (only in all patients), age, TNM stage.

***^b^*** Models were adjusted by sex (only in all patients), age, TNM stage, smoking status, alcohol consumption, body mass index and chemotherapy.
