# Supplementary material for: Association Between Serum Creatinine Concentrations and Overall Survival in Patients With Colorectal Cancer: A Multi-Center Cohort Study
Source: Front Oncol. 2021 Oct 7;11:710423. doi: 10.3389/fonc.2021.710423 (PMC8529284; doi:10.3389/fonc.2021.710423)
Supplement: Supplementary file 3 [file Table_2.docx]

**Supplemental table 2 Association between** **Scr concentrations and OS in patients with CRC (died within one year).**

| Characteristics | Patients (n) | Adjusted HR (95% CI) *^a^* | *P* value | Adjusted HR (95% CI) *^b^* | *P* value |
| --- | --- | --- | --- | --- | --- |
| Scr of all patients |  |  |  |  |  |
| Low *^c^* | 139 | 1.44 (1.10, 1.90) | 0.009 | 1.42 (1.08, 1.88) | 0.013 |
| Normal *^d^* | 138 | Reference |  | Reference |  |
| High *^e^* | 31 | 1.17 (0.73, 1.86) | 0.517 | 1.22 (0.76, 1.96) | 0.415 |
| Scr of men |  |  |  |  |  |
| Low *^c^* | 67 | 1.42 (1.01, 2.01) | 0.049 | 1.38 (0.96, 1.98) | 0.078 |
| Normal *^d^* | 92 | Reference |  | Reference |  |
| High *^e^* | 21 | 1.42 (0.83, 2.43) | 0.197 | 1.53 (0.88, 2.65) | 0.129 |
| Scr of women |  |  |  |  |  |
| Low *^c^* | 72 | 1.33 (0.83, 2.12) | 0.228 | 1.15 (0.90, 1.48) | 0.262 |
| Normal *^d^* | 46 | Reference |  | Reference |  |
| High *^e^* | 10 | 0.65 (0.24, 1.74) | 0.388 | 1.54 (0.96, 2.47) | 0.076 |

Notes:

Abbreviations: Scr, serum creatinine; OS, overall survival; CRC, colorectal cancer; HR, hazard ratio; CI, confidence interval.

***^a^*** Models were adjusted by sex (only in all patients), age, TNM stage.

***^b^*** Models were adjusted by sex (only in all patients), age, TNM stage, smoking status, alcohol consumption, body mass index and chemotherapy.

***^c^*** Low: Scr levels <71 μmol/L in men and <59 μmol/L in women.

***^d^*** Normal: Scr levels ≥71 and ≤104 μmol/L in men and ≥59 and ≤85 μmol/L in women.

***^e^*** High: Scr levels >104 μmol/L in men and >85 μmol/L in women.
