# Supplementary material for: Association Between Serum Creatinine Concentrations and Overall Survival in Patients With Colorectal Cancer: A Multi-Center Cohort Study
Source: Front Oncol. 2021 Oct 7;11:710423. doi: 10.3389/fonc.2021.710423 (PMC8529284; doi:10.3389/fonc.2021.710423)
Supplement: Supplementary file 4 [file Table_3.docx]

**Supplemental table 3 Association between Scr concentrations and OS in patients with CRC (died after one year).**

| Characteristics | Patients (n) | Adjusted HR (95% CI) *^a^* | *P* value | Adjusted HR (95% CI) *^b^* | *P* value |
| --- | --- | --- | --- | --- | --- |
| Scr of all patients |  |  |  |  |  |
| Low *^c^* | 562 | 1.11 (0.87, 1.43) | 0.405 | 1.15 (0.90, 1.48) | 0.262 |
| Normal *^d^* | 784 | Reference |  | Reference |  |
| High *^e^* | 78 | 1.47 (0.92, 2.37) | 0.110 | 1.55 (0.94, 2.55) | 0.076 |
| Scr of men |  |  |  |  |  |
| Low *^c^* | 283 | 1.04 (0.76, 1.43) | 0.789 | 1.09 (0.79, 1.50) | 0.590 |
| Normal *^d^* | 525 | Reference |  | Reference |  |
| High *^e^* | 46 | 1.42 (0.76, 2.66) | 0.272 | 1.51 (0.80, 2.84) | 0.201 |
| Scr of women |  |  |  |  |  |
| Low *^c^* | 279 | 1.22 (0.80, 1.85) | 0.367 | 1.17 (0.76, 1.80) | 0.478 |
| Normal *^d^* | 259 | Reference |  | Reference |  |
| High *^e^* | 32 | 1.63 (0.78, 3.38) | 0.193 | 1.73 (0.83, 3.58) | 0.143 |

Notes:

Abbreviations: Scr, serum creatinine; OS, overall survival; CRC, colorectal cancer; HR, hazard ratio; CI, confidence interval.

***^a^*** Models were adjusted by sex (only in all patients), age, TNM stage.

***^b^*** Models were adjusted by sex (only in all patients), age, TNM stage, smoking status, alcohol consumption, body mass index and chemotherapy.

***^c^*** Low: Scr levels <71 μmol/L in men and <59 μmol/L in women.

***^d^*** Normal: Scr levels ≥71 and ≤104 μmol/L in men and ≥59 and ≤85 μmol/L in women.

***^e^*** High: Scr levels >104 μmol/L in men and >85 μmol/L in women.
